# Long-interval intracortical inhibition in primary motor cortex related to working memory in middle-aged adults

**DOI:** 10.3389/fpsyg.2022.998062

**Published:** 2022-09-28

**Authors:** María Redondo-Camós, Gabriele Cattaneo, Vanessa Alviarez-Schulze, Selma Delgado-Gallén, Goretti España-Irla, Javier Solana-Sanchez, Ruben Perellón-Alfonso, Sergiu Albu, José M. Tormos, Alvaro Pascual-Leone, David Bartres-Faz

**Affiliations:** ^1^Institut Guttmann, Institut Universitari de Neurorehabilitació adscrit a la Universitat Autònoma de Barcelona, Barcelona, Spain; ^2^Departament de Medicina, Facultat de Medicina, Universitat Autònoma de Barcelona, Barcelona, Spain; ^3^Fundació Institut d’Investigació en Ciències de la Salut Germans Trias i Pujol, Barcelona, Spain; ^4^Departamento de Ciencias del Comportamiento, Escuela de Psicología, Universidad Metropolitana, Caracas, Venezuela; ^5^Departament de Medicina, Facultat de Medicina i Ciències de la Salut, i Institut de Neurociències, Universitat de Barcelona, Barcelona, Spain; ^6^Institut d’Investigacions Biomèdiques August Pi i Sunyer (IDIBAPS), Barcelona, Spain; ^7^Hinda and Arthur Marcus Institute for Aging Research and Deanna and Sidney Wolk Center for Memory Health, Hebrew SeniorLife, Boston, MA, United States; ^8^Department of Neurology, Harvard Medical School, Boston, MA, United States

**Keywords:** transcranial magnetic stimulation, electromyography, long-interval cortical inhibition, motor cortex, cognition, resting motor threshold

## Abstract

**Introduction:**

Excitability of the primary motor cortex measured with TMS has been associated with cognitive dysfunctions in patient populations. However, only a few studies have explored this relationship in healthy adults, and even fewer have considered the role of biological sex.

**Methods:**

Ninety-seven healthy middle-aged adults (53 male) completed a TMS protocol and a neuropsychological assessment. Resting Motor Threshold (RMT) and Long-Interval Intracortical Inhibition (LICI) were assessed in the left motor cortex and related to attention, episodic memory, working memory, reasoning, and global cognition composite scores to evaluate the relationship between cortical excitability and cognitive functioning.

**Results:**

In the whole sample, there was a significant association between LICI and cognition; specifically, higher motor inhibition was related to better working memory performance. When the sample was broken down by biological sex, LICI was only associated with working memory, reasoning, and global cognition in men. No associations were found between RMT and cognitive functions.

**Conclusion:**

Greater intracortical inhibition, measured by LICI, could be a possible marker of working memory in healthy middle-aged adults, and biological sex plays a critical role in this association.

## Introduction

The balance of cortical excitation and inhibition (E/I balance) is a core neurophysiologic metric of neuronal and brain network activity believed to determine optimal brain functioning (Sukenik et al., 2021). In patient populations, including autism spectrum disorders or schizophrenia, an E/I imbalance has been observed in different cortical areas and shown to be associated with behavioral and cognitive symptoms (Sohal and Rubenstein, 2019; Bruining et al., 2020; Calvin and Redish, 2021; Maestú et al., 2021). In Alzheimer’s disease, cortical motor hyperexcitability has been negatively related to cognitive performance (Zadey et al., 2021), possibly due to enhanced intracortical excitatory circuits (Di Lazzaro et al., 2004; Meder et al., 2021) or/and an inhibitory deficit (Khedr et al., 2011; Pennisi et al., 2011; Joseph et al., 2021; Mimura et al., 2021).

Past research exploring cortical excitability and cognition has produced inconsistent results in healthy, cognitive-unimpaired adults. This variability may be due to differences depending on the cortical area or specific cognitive functions assessed. For example, while higher excitability after stimulation of the left prefrontal cortex has been related to better executive functions and working memory (Redondo-Camós et al., 2022), other studies have observed that excessive excitability of the primary motor cortex was associated with impaired attention and global cognition (Bolden et al., 2017; Akilan et al., 2020). Other aspects that may modulate the association between cortical E/I balance and cognitive functioning may relate to age, given the different degrees of preservation of gamma-aminobutyric acid (GABA) circuits linked to physiological aging (McGinley et al., 2010; Opie and Semmler, 2014; Hermans et al., 2018). Indeed, age-related cortical excitability changes have been previously linked to differences in attention and inhibitory control in healthy adults (Cespón et al., 2022). Also, biological sex could play a key role since differences in brain anatomy and connectivity between men and women, as well as hormonal influences associated with menstrual cycle variations in women, may lead to distinct neural processes involved in cognitive and motor control (Korzhyk et al., 2019; Rezzani et al., 2019). Gender-associated differences could be due to genetic determinants, lifestyle factors including physical activity, alcohol, or tobacco consumption (Rezzani et al., 2019; Travica et al., 2020), or steroid hormone levels, which are higher in women and have been related to GABA neurotransmission, mood and memory (Cosgrove et al., 2007; Rezzani et al., 2019).

Transcranial Magnetic Stimulation (TMS) combined with Electromyography (EMG) is a widely used technique to study inhibitory and excitatory mechanisms in the motor cortex (Kobayashi and Pascual-Leone, 2003; Ferreri and Rossini, 2013). Specifically, single-pulse TMS (spTMS) has been used to explore cortical excitability by measuring Resting Motor Threshold (RMT), which is the minimum intensity that elicits a Motor Evoked Potential (MEP) of more than 50 μV in 50% of trials (Rossini et al., 2015). Long-Interval Intracortical Inhibition (LICI), where two suprathreshold stimuli separated by an interstimulus interval (ISI) between 50 and 200 ms are applied, has been used to study cortical inhibition (Valls-Solé et al., 1992; Nakamura et al., 1997) and reflects the activity of GABA-B receptors (McDonnell et al., 2006; Opie et al., 2017). While many studies have shown that RMT and LICI offer valuable biological markers in different neurological disorders (Fatih et al., 2021; Guerra et al., 2021; Mimura et al., 2021; Versace et al., 2021), only a few have explored their association with brain health and cognitive performance in healthy, cognitive-unimpaired, middle-aged adults, and how biological sex affects the results (Schicktanz et al., 2014; Akilan et al., 2020).

This study aimed to fill this knowledge gap by investigating the relationship between E/I balance in the primary motor cortex measured with RMT and LICI and cognition in healthy middle-aged adults. Since the GABA-B receptor might be a target for improving cognitive dysfunction and memory/learning impairment (Vlachou, 2022), we hypothesized that better cognitive performance would be associated with more intracortical inhibition, estimated with LICI, and reduced cortical excitability, measured with RMT. Also, differences between women and men were expected, at least for LICI, considering biological sex differences in GABA neurotransmitters highlighted above.

## Materials and methods

### Subjects and study design

**Figure 1:**
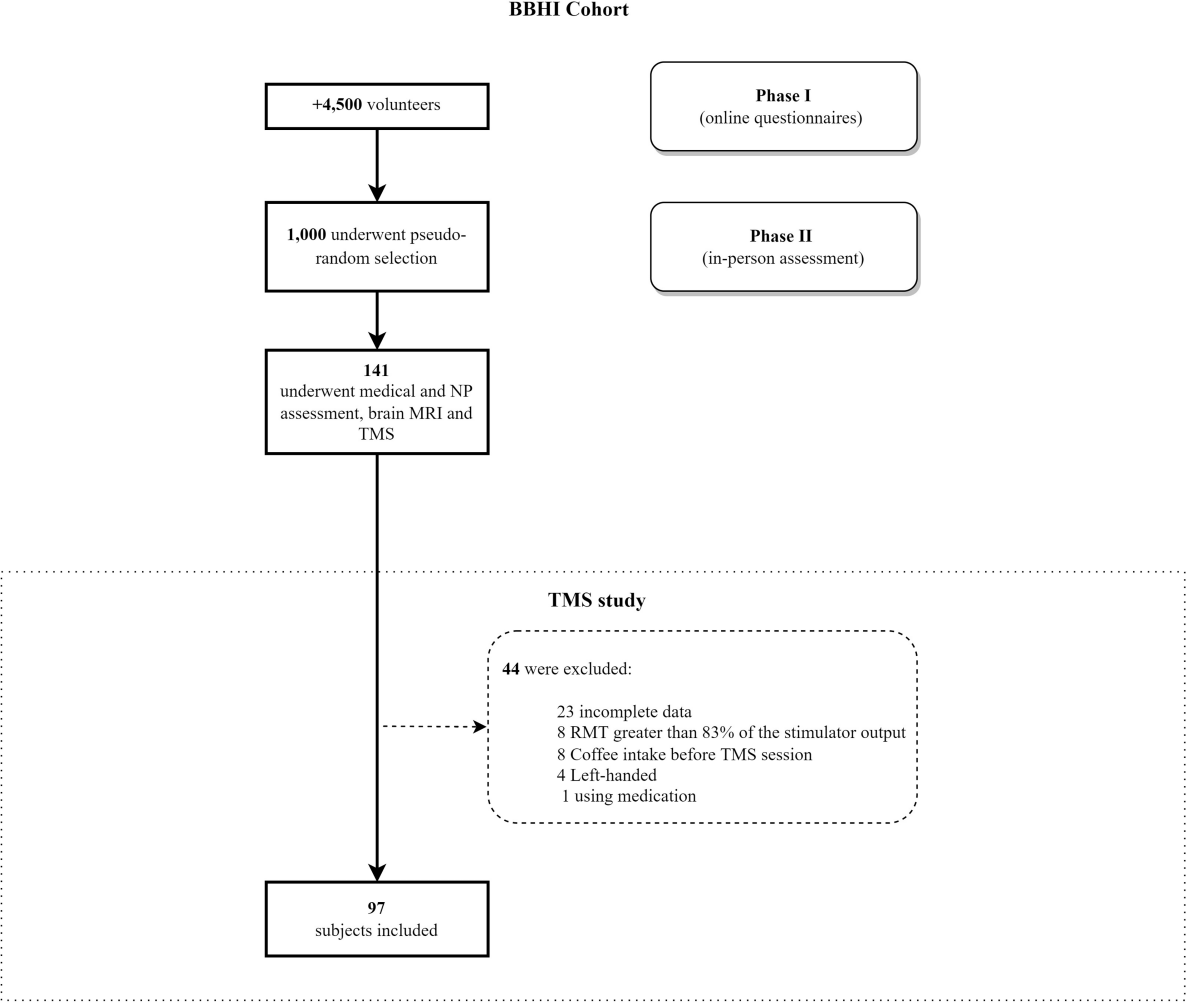
Flowchart of the participant selection for the current analysis. BBHI methodology was used to select volunteers (see Cattaneo et al., 2018, 2020). BBHI, Barcelona Brain Health Initiative; NP, Neuropsychological assessment; MRI, Magnetic resonance imaging; RMT, Resting Motor Threshold.

Ninety-seven healthy and right-handed volunteers [53 male; laterality ≥75%, (Oldfield, 1971)], between 41 and 65 years (M = 54; SD = 7.14), participated in this study. They were part of the Barcelona Brain Health Initiative (BBHI), an ongoing, longitudinal cohort study (Cattaneo et al., 2018). They underwent a TMS session with EMG registration and neuropsychological testing. Exclusion criteria included any neurological or psychiatric diagnosis, currently taking medication that could affect the central nervous system, substance abuse or dependence (alcohol, caffeine, drugs), pregnancy (Rossini et al., 2015; Rossi et al., 2021), and any contraindication for TMS or magnetic resonance imaging (MRI). All participants gave written informed consent, and the local ethics committee (Comité d’Ètica i Investigació Clínica de la Unió Catalana d’Hospitals) approved the study protocol, which followed the Declaration of Helsinki. A cohort diagram from the BBHI study and the specific selection of this study participants is shown in Figure 1.

### TMS protocol

Participants were asked to sit as still as possible in a comfortable armchair, keep their eyes open, and look at a fixation cross at a distance of approximately 1.5 m. A figure of eight TMS coil was placed at a 45-degree angle (relative to the mid-sagittal plane) over the left primary motor cortex (left-M1), resulting in a posterior-to-anterior current flow. Consistency in the stimulation targeting was ensured using a frameless stereotactic neuronavigation system (Brainsight, Rogue Research Inc., Montreal, QC Canada) guided by each subject’s T1 weighted structural MRI (previously obtained from a 3 T Siemens Magnetom Prisma). MRI was completed for this purpose to increase safety during TMS sessions and exclude any brain lesion that could act as a confounder in interpreting the results.

The TMS procedure lasted approximately 1 h. First, RMT was determined as the minimum TMS intensity that elicited MEPs of more than 50 μV in five out of 10 trials in the relaxed, contralateral first dorsal interosseus muscle (FDI; Rossini et al., 2015). MEP amplitude was defined as the peak-to-peak difference in EMG activity from the evoked response in this muscle. Next, 120 paired-pulse TMS stimuli were delivered to the left-M1 at random intervals between 3 and 6 s. The intensities of both pulses were applied at 120% of RMT, and the ISI was 100 ms, selecting this interval because it was reportedly optimal (Sanger et al., 2001), and previous research has suggested age-related changes at it (McGinley et al., 2010; Opie et al., 2015, 2018). From this stimulation, LICI was calculated using the following formula (Guerra et al., 2021):


$$\mathrm{LICI}=\left(\frac{MEP\;\mathrm{amplitude conditioned stimulus}}{MEP\;\mathrm{amplitude unconditioned stimulus}}\right)*100$$

Consequently, a greater LICI value indicates lower cortical inhibition, while a smaller LICI indicates greater inhibition.

The protocol was completed using a figure-of-eight Cool-B65 coil connected to a Medtronic MagPro X100 stimulator (MagVenture A/S, Denmark). For the electromyography, a Biopac EMG100C amplifier (BIOPAC Systems INC., California, United States) was used with surface electrodes placed in a belly-tendon montage and the ground electrode on the ulnar styloid.

### Neuropsychological assessment

A licensed neuropsychologist performed a battery of neurocognitive paper and pencil evaluations. The battery included the following tests: Trail Making Test A and B (TMT) (Reitan and Wolfson, 1985; Peña-Casanova et al., 2012), Digit-Span Forward and Backward, Corsi block tapping test, Letter-Number Sequencing test (Peña-Casanova et al., 2012), Matrix Reasoning and Block design, the Digit symbol task, the Cancelation test (Wechsler, 2012), the Rey Auditory Verbal Learning Test (RAVLT; Schmidt, 1996; Alviarez-Schulze et al., 2022b), and the Spanish Version of the Face Name Associative Memory Exam (S-FNAME; Alegret et al., 2015; Alviarez-Schulze et al., 2022a).

### Statistical analysis

All statistical analyses were performed in SPSS version 22.0 (Statistical Package for Social Sciences, Chicago, IL, United States).

First, raw scores of each cognitive test were z-score normalized, and principal component analysis (PCA) was run to group them into cognitive domains, in line with our and other research group’s previous studies (España-Irla et al., 2021; Cattaneo et al., 2022; Hinchman et al., 2022; Redondo-Camós et al., 2022). Loading values were above 0.3. Kaiser–Meyer–Olkin (KMO = 0.689) and Bartlett’s test of sphericity (χ^2^ = 1074.67, df = 105; *p* < 0.001) were satisfactory. PCA revealed four components of cognitive domains. The first factor contained TMT B (−0.927), TMT B-A (−0.884), TMT A (−0.615), Digit symbol task (0.554), and cancelation test (0.423), reflecting what can be considered an attentional domain. The second factor characterized memory and involved face name (0.609) and RAVLT measures such as immediate recall (0.885), delayed recall (0.872), and recognition (0.830). The third factor reflected a working memory domain and included the digit forward (0.780), digit backward (0.777), and letter-number sequencing (0.504). The fourth factor contained Block design (0.758), Corsi blocks (0.678), and matrix reasoning (0.620), representing a reasoning component. Ultimately, a global cognition score was created as the sum of the individual z-scores on each neuropsychological test.

Cognitive composite scores were used as dependent variables (attention, working memory, episodic memory, reasoning, and global cognition) and RMT, LICI, MEP amplitude, age, biological sex, and years of education as predictors. We ran multiple multivariate regressions to identify possible associations between motor cortical excitability (measured by RMT), inhibition (LICI), and cognition. Then, for significant results, we ran multiple linear regressions to assess the direction of the prediction. Assumptions of linearity, independence of residuals, homoscedasticity, multicollinearity, and normality were met in all models. Furthermore, to study how biological sex could affect the predictions, we did all the previous analysis segmenting by biological sex. Lastly, to explore possible differences between means of women and men on each variable, a t-test analysis was performed.

## Results

Sample descriptive statistics of RMT, LICI, MEP Amplitude, age, biological sex, and educational level are presented in Table 1, while cognitive scores are in Table 2.

**Table 1:** Demographic Variables, RMT and LICI (*n* = 97).

	All (*n* = 97)		Male (*n* = 53)		Female (*n* = 44)		
	Mean	SD	Mean	SD	Mean	SD	*p*
Age	53.69	7.13	53.49	7.40	53.93	6.87	0.762
Years of education	17.85	3.81	17.66	4.17	18.07	3.35	0.595
RMT (%)	62.28	9.60	61.64	10.54	63.05	8.4	0.467
LICI (%)	14.68	18.81	17.35	20.33	11.46	16.45	0.118
MEP amplitude (mV)	0.305	0.201	0.309	0.173	0.323	0.232	0.747

RMT, resting motor threshold; LICI, long-interval intracortical inhibition; MEP, motor evoked potential.

**Table 2:** Cognitive scores (*n* = 97).

	All (*n* = 97)		Male (*n* = 53)		Female (*n* = 44)		
Cognitive task	Mean	SD	Mean	SD	Mean	SD	*p*
S-FNAME	41.62	14.65	39.00	14.12	44.77	14.81	0.054
RAVLT immediate recall	52.19	9.65	50.60	9.93	54.10	9.06	0.074
RAVLT delayed recall	11.46	2.79	11.04	2.78	11.98	2.74	0.098
RAVLT recognition	14.28	1.25	14.17	1.27	14.41	1.23	0.348
Digit-span forward	10.63	2.86	11.02	2.87	10.16	2.79	0.140
Digit-span backward	11.38	2.60	11.68	2.66	11.02	2.50	0.214
Corsi block tapping	14.35	2.41	14.38	2.51	14.32	2.31	0.904
Letter-number sequencing	5.72	1.08	5.66	1.14	5.80	1.00	0.542
Matrix reasoning WAIS-IV	13.91	2.58	14.32	2.38	13.41	2.75	0.087
Block design WAIS-IV	12.00	3.14	12.74	3.01	11.11	3.10	0.011^*^
TMT A	11.26	2.51	11.28	2.76	11.23	2.22	0.912
TMT B	8.65	2.18	8.91	2.14	8.34	2.22	0.208
Cancelation test	42.09	8.17	42.04	8.67	42.16	7.63	0.942
Digit symbol task	13.84	2.56	13.51	2.49	14.23	2.60	0.171

S-FNAME, Spanish Version of the Face Name Associative Memory Exam; RAVLT immediate recall, Recall a list of words immediately after hearing it of Rey Auditory Verbal Learning Test; RAVLT delayed recall, RAVLT recall after 30 min; RAVLT recognition, Recognition of words from a word list of RAVLT; Digit-Span Forward, Immediate recall a series of numbers in the same order; Letter-Number Sequencing, Sequence a random order of numbers and letter; Matrix Reasoning WAIS-IV, Logical sequences and series of Wechsler Adult Intelligence Scale-IV; Block Design WAIS-IV, Block Design of Wechsler Adult Intelligence Scale-IV; TMT B, Trail Making Test part B; TMT A, Trail Making Test part A; Digit symbol task, Digit symbol association; Cancelation test, cancelation task of WAIS-IV. All punctuations presented in this table are normalized scores except RAVLT tests that are raw scores.

*p < 0.05.

### Associations between RMT, LICI, and cognitive functions

Multivariate regression analysis for all the subjects revealed statistically significant associations between LICI and working memory [*F*(1, 89) = 7.59, *p* = 0.007; partial η2 = 0.079], and biological sex and episodic memory [*F*(1, 89) = 9.50, *p* = 0.003; partial η2 = 0.0996] and reasoning [*F*(1, 89) = 4.26, *p* = 0.042; partial η2 = 0.046]. Finally, age was significantly associated to all cognitive functions (attention [F (1, 89) = 17.38, *p* < 0.001; partial η2 = 0.163], episodic memory [*F*(1, 89) = 10.01, *p* = 0.002; partial η2 = 0.101], working memory [*F*(1, 89) = 5.45, *p* = 0.022; partial η2 = 0.058], reasoning [*F*(1, 89) = 6.66, *p* = 0.011; partial η2 = 0.070], and global cognition [*F*(1, 89) = 14.50, *p* < 0.001; partial η2 = 0.140]).

**Figure 2:**
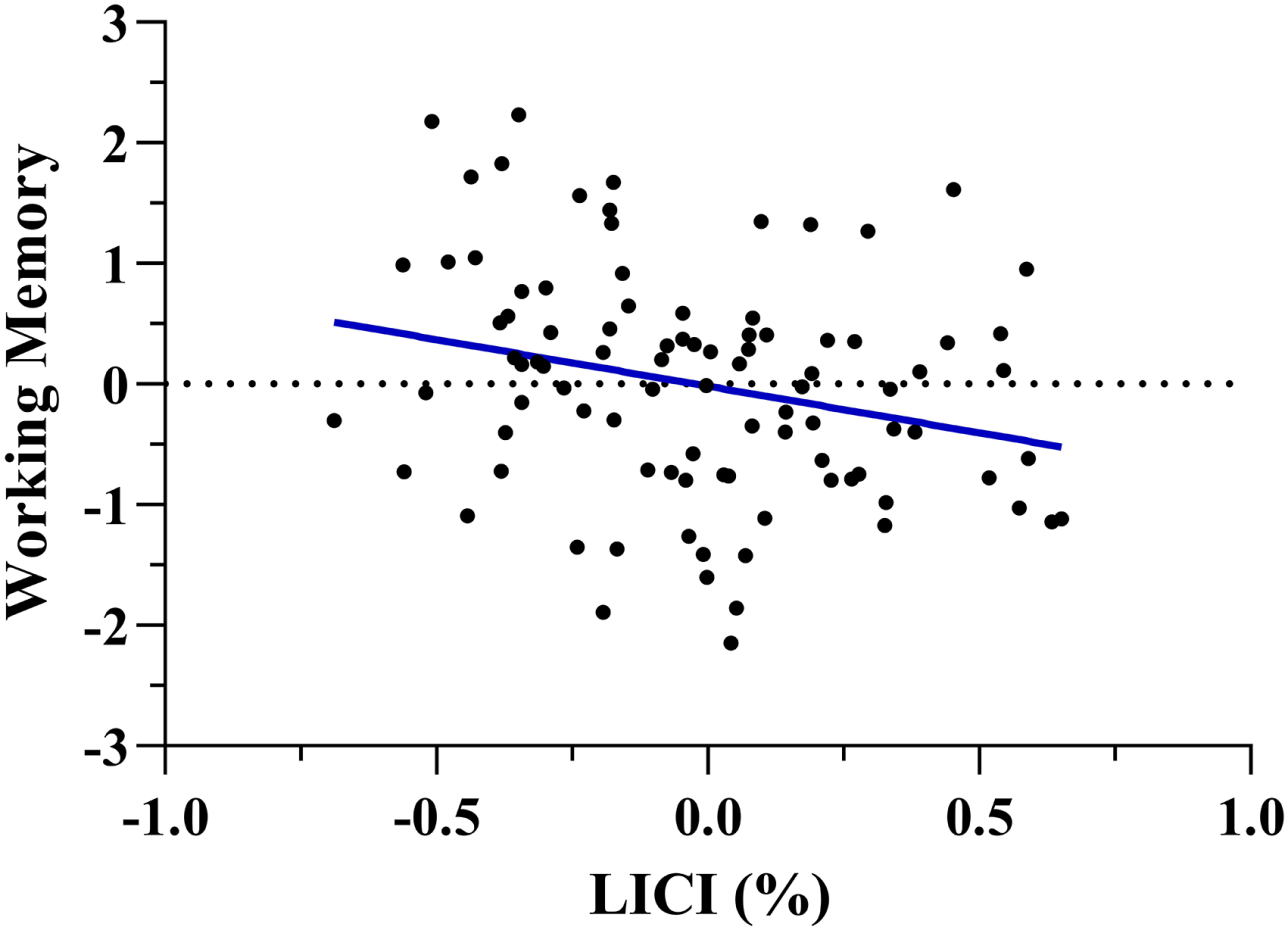
Multiple regression scatterplots between LICI and working memory after controlling age, biological sex, and years of education. Z-scores were used on the Y-axis and unstandardized Predicted Values on the X-axis (%). A lower LICI value, indicative of more intracortical motor inhibition, was related to better working memory performance.

After running this model, we ran a multiple regression to assess the direction of the prediction using the working memory domain as a dependent variable and LICI, RMT, MEP amplitude, age, biological sex, and education as regressors. The model significantly explained working memory performance, [*F*(6, 89) = 2.630, *p* = 0.021, adj. R^2^ = 0.093], and LICI resulted negatively associated with it (Standardized β = −0.282, t = −2.754, *p* = 0.007), being greater motor cortical inhibition related to better working memory (Figure 2).

### Effect of biological sex

To investigate possible differences in the association between cognitive performance and cortical E/I, we split the sample according to biological sex and repeated regressions for both groups.

#### Men

Multivariate regressions were done to test the direct effects of RMT, LICI, MEP amplitude, and covariates on each cognitive domain. They revealed an association between LICI and working memory [*F*(1, 47) = 6.60, *p* = 0.013, partial η2 = 0.123], reasoning [*F*(1, 47) = 5.82, *p* = 0.020, partial η2 = 0.110], and global cognition [*F*(1, 47) = 9.22, *p* = 0.004, partial η2 = 0.164]. Also, education was related to reasoning [*F*(1, 47) = 6.02, *p* = 0.018, partial η2 = 0.114] and episodic memory [*F*(1, 47) = 4.15, *p* = 0.047, partial η2 = 0.081], and age to all cognitive domains (attention [*F*(1, 47) = 4.18, *p* = 0.047; partial η2 = 0.082], episodic memory [*F*(1, 47) = 8.37, *p* = 0.006; partial η2 = 0.151], working memory [*F*(1, 47) = 4.64, *p* = 0.03; partial η2 = 0.090], reasoning [*F*(1, 47) = 9.01, *p* = 0.004; partial η2 = 0.161], and global cognition [*F*(1, 47) = 7.40, *p* = 0.009; partial η2 = 0.136]).

**Figure 3:**
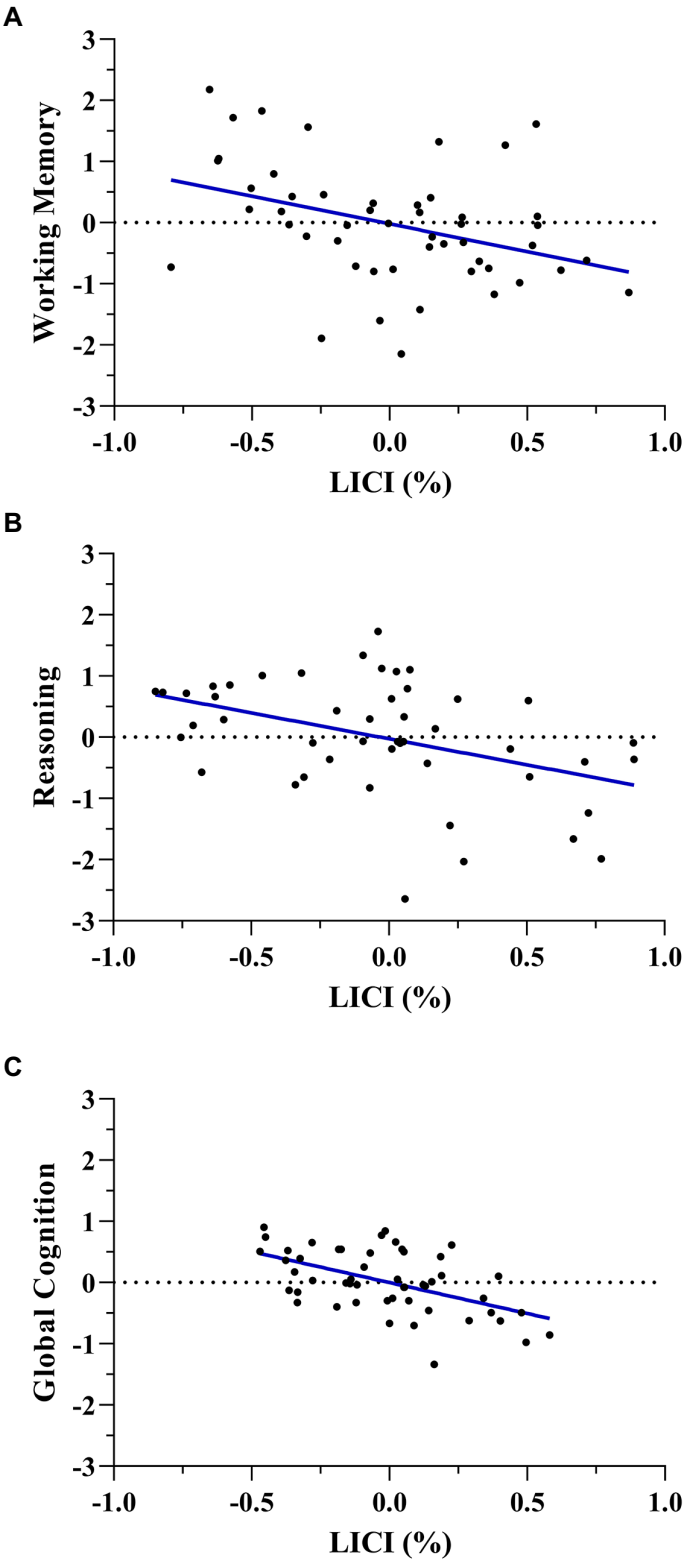
Multiple regression scatterplots between LICI and working memory **(A)**, reasoning **(B)**, and global cognition **(C)** after controlling age and years of education in men. Z-scores were used on the Y-axis and unstandardized Predicted Values on the X-axis (%).

Furthermore, multiple regression models including covariates showed that working memory was associated with LICI (Standardized β = −0.347, t = −2.569, *p* = 0.013) and age (Standardized β = 0.284, t = 2.155, *p* = 0.036). Also, reasoning was related to LICI (Standardized β = −0.302, t = −2.411, *p* = 0.020), age (Standardized β = −0.367, t = −3.022, *p* = 0.004) and education level (Standardized β = 0.307, t = 2.454, *p* = 0.018). Finally, global cognition was also associated with LICI (Standardized β = −0.380, t = −3.036, *p* = 0.004) and age (Standardized β = −0.332, t = −2.721, *p* = 0.009). In all three models, LICI was negatively associated with working memory, reasoning, and global cognition in men (Figure 3).

#### Women

Only were significant associations between age and attention [*F*(1, 37) = 11.65, *p* = 0.002; partial η2 = 0.240]. No significant results were seen between women’s cognition and cortical excitability or inhibition.

## Discussion

The current study explored the relationship between cortical measures of E/I balance in the primary motor cortex, using TMS measures of RMT and LICI, and cognitive performance in healthy, cognitively-unimpaired middle-aged adults. Moreover, we studied the impact of biological sex on this association. Our results reveal that lower LICI in the motor cortex is associated with better working memory performance in the whole sample. The effect appears primarily accounted for by men, in whom LICI was found to be related to increased working memory, reasoning, and global cognition. RMT was not associated with cognitive functions in men or women.

Our results are in line with previous research investigating physiological mechanisms of neurological disorders, showing that motor hyperexcitability is related to global cognitive dysfunction (Takahashi et al., 2013; Higashihara et al., 2021; Zadey et al., 2021) due to increased excitatory activity or/and an inhibitory deficit (Joseph et al., 2021; Meder et al., 2021; Mimura et al., 2021). Similarly, in healthy subjects, it has been found that hyperexcitability of the motor cortex is associated with impaired attention (Bolden et al., 2017; Akilan et al., 2020), suggesting that cortical excitatory and inhibitory balance is necessary for optimal brain and cognitive functioning (Páscoa dos Santos and Verschure, 2022).

The relationship between cognition and motor cortex activity could result from the functional connectivity between brain regions involved in cognitive processing (Bates and Goldman-Rakic, 1993; Hasan et al., 2013). For example, the prefrontal cortex is essential for the performance of higher cognitive functions, and the perturbation of its structure or functionality, such as occurs in aging or Alzheimer’s disease (Salat et al., 2001; Peters, 2006), could alter the cortical excitability of it (Noda et al., 2017) and highly connected areas such as motor cortex (Freeman et al., 2016). These areas could share evolutive roots, and their interaction is needed to govern the executive function and the intentionality of movements (Mendoza and Merchant, 2014; Leisman et al., 2016). Working memory (Carruthers, 2013; Liao et al., 2014; Leisman et al., 2016), attention, and learning (Bhattacharjee et al., 2021) are some of the cognitive functions that have been related to motor processes.

Interestingly, only intracortical motor inhibition was positively associated with cognitive performance, particularly working memory, necessary to serve other cognitive functions (Mansouri et al., 2015), and defined as a limited capacity system allowing the temporary storage and manipulation of information required for such complex processes (Baddeley, 2000). Indeed, we found that LICI was also related to reasoning and global cognition in men, possibly because working memory could play a role in these cognitive functions (Hambrick and Engle, 2003; Wiley and Jarosz, 2012). Given that in our study, intracortical motor inhibition was measured using the LICI paradigm that reflects GABA-B inhibitory neurotransmission (Valls-Solé et al., 1992), we believe that its alteration was associated with cognitive changes. GABA-B receptor indeed has been previously linked to memory formation (Terunuma et al., 2014; Almasi et al., 2018) and working memory (Bañuelos et al., 2014; Schmidt-Wilcke et al., 2018). Furthermore, Freeman et al. (2016) found increased intracortical inhibition in the motor cortex under high working memory load tasks, indicating an association between it and the balance of excitatory/inhibitory activity. These results demonstrate that working memory, even if it strongly involves prefrontal cortex activity, depending on the task’s difficulty, requires motor inhibition to work efficiently (Freeman et al., 2016). It was also supported by the results of Liao et al. (2014), which demonstrated the specific involvement of the primary motor cortex and the motor network in working memory processing (Liao et al., 2014).

Specifically, the psychometric measures included in the working memory domain are exclusively auditory-verbal tests. Within the multicomponent working memory model (Baddeley, 2000), one of the most cited in the literature (Chai et al., 2018), our study data reflect the phonological loop subcomponent, responsible for holding verbal information using a temporary store and an articulatory rehearsal system, and the central executive subcomponent responsible for the active manipulation (serial ordering) of the information. Previous findings have shown a double anatomical dissociation in which the subprocess of temporal retention of verbal information depends predominantly on the superior temporal gyrus. However, the subprocess of information manipulation in backward items with higher cognitive load involves, in addition to, the prefrontal area, motor, and somatosensory cortex (Ghaleh et al., 2020).

Furthermore, when we split the sample for biological sex, we observed that women presented subtly higher inhibition than men, which was not statistically significant. This result is in line with previous studies showing that inhibition, usually superior in women, could be influenced by different functional brain maturation of the inhibitory system (Rubia et al., 2013), variations of the brain areas activated (Bell et al., 2006; Li et al., 2006; Korzhyk et al., 2019), and ovarian hormones (Hosseini-Kamkar and Bruce Morton, 2014; Shibuya et al., 2016).

Crucially, we found a positive association between cognition (working memory and reasoning) and intracortical inhibition only in men. Very little is present in the literature on this issue, and the few existing pieces of evidence appear somehow contradictory. Schicktanz et al. (2014) observed that lower motor cortical excitability was related to better working memory in men (Schicktanz et al., 2014), while Akilan et al. (2020) observed that an increase in cortical excitability was related to global cognition in women (Akilan et al., 2020). However, beyond these inconsistencies, which need and deserve deeper study, previous and our results confirm the existence of biological sex differences (Schicktanz et al., 2014; Akilan et al., 2020) in the relation between cortical excitability/inhibition and cognition.

This research increases our knowledge of this association and suggests that greater intracortical inhibition, measured by LICI, is a possible marker of interindividual differences in working memory performance among healthy middle-aged adults, extending previous suggestions as a biomarker of neuropsychiatric disorders (Fatih et al., 2021).

Finally, the findings in this report are subject to some limitations. First, this is a cross-sectional study, and we cannot determine a cause-effect relationship between cortical excitability measures and cognitive performance. It is necessary to deepen in future research the relationship described in our study, including visuospatial working memory tests and other tasks with different levels of cognitive load and, within the manipulation process, not only serial ordering but updating processing, such as n-back paradigm. Defining which specific working components are related to the TMS measure studied is essential. Also, these processes differ in their sensitivity to advancing age (Jablonska et al., 2020); hence, future investigations should be conducted in other age groups, including middle and older age samples. Furthermore, we explored two TMS measures (RMT and LICI), but it would be interesting to consider other LICI ISI (50 ms, 150 ms, and 200 ms) and paradigms such as short-interval intracortical inhibition or intracortical facilitation. Ultimately, emotional state, sleep quality, and menstrual cycle variations (in particular when considering younger women populations) should be considered in future analysis, being this latter one relevant due to its impact on the female brain and needing specific investigations on this gender group (Hidalgo-Lopez et al., 2020; Meeker et al., 2020).

## Data availability statement

The raw data supporting the conclusions of this article will be made available by the authors, without undue reservation.

## Ethics statement

The studies involving human participants were reviewed and approved by Comité d’Ètica i Investigació Clínica de la Unió Catalana d’Hospitals. The patients/participants provided their written informed consent to participate in this study.

## Author contributions

AP-L, DB-F, and JT participated in the initial conception of the design of the BBHI project. MR-C, DB-F, AP-L, and GC contributed to the conception and design of the present study. MR-C, SD-G, GE-I, VA-S, SA, RP-A, and JS-S contributed to the data acquisition. MR-C and GC analyzed the data. MR-C, GC, and DB-F contributed to the first draft of the manuscript. All authors contributed to the article and approved the submitted version.

## Funding

DB-F and RP-A were funded by a Spanish Ministry of Science, Innovation and Universities (MICIU/FEDER; RTI2018-095181-B-C21) and also supported by an ICREA Academia 2019 grand award. RP-A was supported by a fellowship from “la Caixa” Foundation (ID 100010434, Fellowship code: LCF/BQ/DI19/11730050), JT was partly supported Fundació Joan Ribas (Araquistain_FJRA), AGAUR, Agència de Gestió d’Ajuts Universitaris i de Recerca. Convocatòria 2018 d’Indústria del Coneixement (modalitat PRODUCTE) and FEDER, Fons Europeu de Desenvolupament Regional (2018 PROD 00172), Fundació La Marató De TV3 (201735.10), and European Commission—H2020/Call H2020-SC1-2016-2017 (RIA; grant agreement no. 777107). AP-L was partly supported by the National Institutes of Health (R24AG06142 and P01 AG031720) and the Barcelona Brain Health Initiative funded primarily by “La Caixa” (LCF/PR/PR16/11110004). The funders were not involved in the study design, collection, analysis, interpretation of data, the writing of this article or the decision to submit it for publication.

## Conflict of interest

AP-L is a co-founder of Linus Health and TI Solutions AG; serves on the scientific advisory boards for Starlab Neuroscience, Magstim Inc., Radiant Hearts, Skin2Neuron, TetraNeuron, and MedRhythms; is listed as an inventor on several issued and pending patents on the real-time integration of noninvasive brain stimulation with electroencephalography and magnetic resonance imaging.

The remaining authors declare that the research was conducted in the absence of any commercial or financial relationships that could be construed as a potential conflict of interest.

## Publisher’s note

All claims expressed in this article are solely those of the authors and do not necessarily represent those of their affiliated organizations, or those of the publisher, the editors and the reviewers. Any product that may be evaluated in this article, or claim that may be made by its manufacturer, is not guaranteed or endorsed by the publisher.

## References

[ref1] Akilan K. Kumar S. Zomorrodi R. Blumberger D. M. Daskalakis Z. J. Rajji T. K. (2020). Gender impact on transcranial magnetic stimulation-based cortical excitability and cognition relationship in healthy individuals. Neuroreport 31, 287–292. doi: 10.1097/WNR.0000000000001392, PMID: 31895750

[ref2] Alegret M. Valero S. Ortega G. Espinosa A. Sanabria A. Hernández I.. (2015). Validation of the Spanish version of the face name associative memory exam (S-FNAME) in cognitively normal older individuals. Arch. Clin. Neuropsychol. 30, 712–720. doi: 10.1093/arclin/acv050, PMID: 26289054 PMC4751234

[ref3] Almasi A. Zarei M. Raoufi S. Sarihi A. Salehi I. Komaki A.. (2018). Influence of hippocampal GABAB receptor inhibition on memory in rats with acute β-amyloid toxicity. Metab. Brain Dis. 33, 1859–1867. doi: 10.1007/s11011-018-0292-5, PMID: 30039187

[ref4] Alviarez-Schulze V. Cattaneo G. Pachón-García C. Solana-Sánchez J. Tormos J. M. Pascual-Leone A.. (2022b). Validation and normative data of the Spanish version of the Rey auditory verbal learning test and associated long-Term forgetting measures in middle-aged adults. Front. Aging Neurosci. 14:809019. doi: 10.3389/fnagi.2022.809019, PMID: 35221995 PMC8865334

[ref5] Alviarez-Schulze V. Cattaneo G. Pachón-García C. Solana-Sánchez J. Tormos-Muñoz J. M. Alegret M.. (2022a). Validation and normative data of the Spanish version of the face name associative memory exam (S-FNAME). J. Int. Neuropsychol. Soc. 28, 74–84. doi: 10.1017/S1355617721000084, PMID: 33749568

[ref6] Baddeley A. (2000). The episodic buffer: a new component of working memory? *Trends Cogn*. Science 4, 417–423. doi: 10.1016/S1364-6613(00)01538-2, PMID: 11058819

[ref7] Bañuelos C. Sofia Beas B. McQuail J. A. Gilbert R. J. Frazier C. J. Setlow B.. (2014). Prefrontal cortical GABAergic dysfunction contributes to age-related working memory impairment. J. Neurosci. 34, 3457–3466. doi: 10.1523/JNEUROSCI.5192-13.2014, PMID: 24599447 PMC3942567

[ref500] Bates J. F. Goldman-Rakic P. S. (1993). Prefrontal connections of medial motor areas in the rhesus monkey. J. Comp. Neurol. 336, 211–228. doi: 10.1002/cne.903360205, PMID: 7503997

[ref8] Bell E. C. Willson M. C. Wilman A. H. Dave S. Silverstone P. H. (2006). Males and females differ in brain activation during cognitive tasks. Neuroimage 30, 529–538. doi: 10.1016/j.neuroimage.2005.09.049 16260156

[ref9] Bhattacharjee S. Kashyap R. Abualait T. Annabel Chen S. H. Yoo W. K. Bashir S. (2021). The role of primary motor cortex: more than movement execution. J. Mot. Behav. 53, 258–274. doi: 10.1080/00222895.2020.1738992, PMID: 32194004

[ref10] Bolden L. B. Griffis J. C. Pati S. Szaflarski J. P. (2017). Cortical excitability and neuropsychological functioning in healthy adults. Neuropsychologia 102, 190–196. doi: 10.1016/j.neuropsychologia.2017.06.028, PMID: 28648572

[ref11] Bruining H. Hardstone R. Juarez-Martinez E. L. Sprengers J. Avramiea A. E. Simpraga S.. (2020). Measurement of excitation-inhibition ratio in autism spectrum disorder using critical brain dynamics. Sci. Rep. 10, 9195–9115. doi: 10.1038/s41598-020-65500-4, PMID: 32513931 PMC7280527

[ref12] Calvin O. L. Redish A. D. (2021). Global disruption in excitation-inhibition balance can cause localized network dysfunction and schizophrenia-like context-integration deficits. PLoS Comput. Biol. 17:e1008985. doi: 10.1371/journal.pcbi.1008985, PMID: 34033641 PMC8184155

[ref13] Carruthers P. (2013). Evolution of working memory. Proc. Natl. Acad. Sci. U. S. A. 110, 10371–10378. doi: 10.1073/PNAS.1301195110, PMID: 23754428 PMC3690618

[ref14] Cattaneo G. Bartrés-Faz D. Morris T. P. Sánchez J. S. Macià D. Tarrero C.. (2018). The Barcelona brain health initiative: a cohort study to define and promote determinants of brain health. Front. Aging Neurosci. 10:321. doi: 10.3389/fnagi.2018.00321, PMID: 30405394 PMC6204574

[ref15] Cattaneo G. Bartrés-Faz D. Morris T. P. Sánchez J. S. Macià D. Tormos J. M.. (2020). The Barcelona brain health initiative: cohort description and first follow-up. PLoS One 15:e0228754. doi: 10.1371/JOURNAL.PONE.0228754, PMID: 32045448 PMC7012435

[ref16] Cattaneo G. Solana-Sánchez J. Abellaneda-Pérez K. Portellano-Ortiz C. Delgado-Gallén S. Alviarez Schulze V.. (2022). Sense of coherence mediates the relationship between cognitive reserve and cognition in middle-aged adults. Front. Psychol. 13:835415. doi: 10.3389/fpsyg.2022.835415, PMID: 35418913 PMC8996461

[ref17] Cespón J. Pellicciari M. C. Casula E. P. Miniussi C. (2022). Age-related changes in cortical excitability linked to decreased Attentional and inhibitory control. Neuroscience 495, 1–14. doi: 10.1016/j.neuroscience.2022.05.021, PMID: 35605905

[ref18] Chai W. J. Abd Hamid A. I. Abdullah J. M. (2018). Working memory from the psychological and neurosciences perspectives: a review. Front. Psychol. 9:401. doi: 10.3389/FPSYG.2018.00401, PMID: 29636715 PMC5881171

[ref19] Cosgrove K. P. Mazure C. M. Staley J. K. (2007). Evolving knowledge of sex differences in brain structure, function, and chemistry. Biol. Psychiatry 62, 847–855. doi: 10.1016/j.biopsych.2007.03.001, PMID: 17544382 PMC2711771

[ref20] Di Lazzaro V. Oliviero A. Pilato F. Saturno E. Dileone M. Marra C.. (2004). Motor cortex hyperexcitability to transcranial magnetic stimulation in Alzheimer's disease. J. Neurol. Neurosurg. Psychiatry 75, 555–559. doi: 10.1136/jnnp.2003.018127, PMID: 15026495 PMC1739006

[ref21] España-Irla G. Gomes-Osman J. Cattaneo G. Albu S. Cabello-Toscano M. Solana-Sanchéz J.. (2021). Associations between cardiorespiratory fitness, cardiovascular risk, and cognition are mediated by structural brain health in midlife. J. Am. Heart Assoc. 10:e020688. doi: 10.1161/JAHA.120.020688, PMID: 34514813 PMC8649552

[ref22] Fatih P. Kucuker M. U. Vande Voort J. L. Doruk Camsari D. Farzan F. Croarkin P. E. (2021). A systematic review of long-interval Intracortical inhibition as a biomarker in neuropsychiatric disorders. Front. Psych. 12:678088. doi: 10.3389/fpsyt.2021.678088, PMID: 34149483 PMC8206493

[ref23] Ferreri F. Rossini P. M. (2013). TMS and TMS-EEG techniques in the study of the excitability, connectivity, and plasticity of the human motor cortex. Rev. Neurosci. 24, 431–442. doi: 10.1515/revneuro-2013-0019, PMID: 23907420

[ref24] Freeman S. M. Itthipuripat S. Aron A. R. (2016). High working memory load increases intracortical inhibition in primary motor cortex and diminishes the motor affordance effect. J. Neurosci. 36, 5544–5555. doi: 10.1523/JNEUROSCI.0284-16.2016, PMID: 27194334 PMC6601763

[ref25] Ghaleh M. Lacey E. H. Fama M. E. Anbari Z. Demarco A. T. Turkeltaub P. E. (2020). Dissociable mechanisms of verbal working memory revealed through multivariate lesion mapping. Cereb. Cortex 30, 2542–2554. doi: 10.1093/CERCOR/BHZ259, PMID: 31701121 PMC7305798

[ref26] Guerra A. Rocchi L. Grego A. Berardi F. Luisi C. Ferreri F. (2021). Contribution of TMS and TMS-EEG to the understanding of mechanisms underlying physiological brain aging. Brain Sci. 11:405. doi: 10.3390/brainsci11030405, PMID: 33810206 PMC8004753

[ref27] Hambrick D. Z. Engle R. W. (2003). “The role of working memory in problem solving,” in The psychology of problem solving. eds. Davidson J. E. Sternberg R. J. (Cambridge: Cambridge University Press), 176–206.

[ref600] Hasan A. Galea J. M. Casula E. P. Falkai P. Bestmann S. Rothwell J. C. (2013). Muscle and timing-specific functional connectivity between the dorsolateral prefrontal cortex and the primary motor cortex. J. Cogn. Neurosci. 25, 558–570. doi: 10.1162/jocn_a_00338, PMID: 23249357 PMC3586373

[ref28] Hermans L. Levin O. Maes C. van Ruitenbeek P. Heise K. F. Edden R. A. E.. (2018). GABA levels and measures of intracortical and interhemispheric excitability in healthy young and older adults: an MRS-TMS study. Neurobiol. Aging 65, 168–177. doi: 10.1016/j.neurobiolaging.2018.01.023, PMID: 29494863

[ref29] Hidalgo-Lopez E. Mueller K. Harris T. A. Aichhorn M. Sacher J. Pletzer B. (2020). Human menstrual cycle variation in subcortical functional brain connectivity: a multimodal analysis approach. Brain Struct. Funct. 225, 591–605. doi: 10.1007/s00429-019-02019-z, PMID: 31894405 PMC7046575

[ref30] Higashihara M. Pavey N. van den Bos M. Menon P. Kiernan M. C. Vucic S. (2021). Association of Cortical Hyperexcitability and Cognitive Impairment in patients with amyotrophic lateral sclerosis. Neurology 96, e2090–e2097. doi: 10.1212/WNL.0000000000011798, PMID: 33827958

[ref31] Hinchman C. A. Cabral D. F. Ciesla M. Flothmann M. Nunez C. Rice J.. (2022). Exercise engagement drives changes in cognition and cardiorespiratory fitness after 8 weeks of aerobic training in sedentary aging adults at risk of cognitive decline. Front. Rehabil. Sci. 3:923141. doi: 10.3389/FRESC.2022.923141 36189006 PMC9397848

[ref32] Hosseini-Kamkar N. Bruce Morton J. (2014). Sex differences in self-regulation: an evolutionary perspective. Front. Neurosci. 8:233. doi: 10.3389/fnins.2014.00233, PMID: 25140126 PMC4121536

[ref33] Jablonska K. Piotrowska M. Bednarek H. Szymaszek A. Marchewka A. Wypych M.. (2020). Maintenance vs. manipulation in auditory verbal working memory in the elderly: new insights based on temporal dynamics of information processing in the millisecond time range. Front. Aging Neurosci. 12:194. doi: 10.3389/FNAGI.2020.00194, PMID: 32848698 PMC7396649

[ref34] Joseph S. Patterson R. Wang W. Blumberger D. M. Rajji T. Kumar S. (2021). Quantitative assessment of cortical excitability in Alzheimer’s dementia and its association with clinical symptoms: a systematic review and meta-analyses. J. Alzheimers Dis. 88, 867–891. doi: 10.3233/jad-210311 34219724

[ref35] Khedr E. M. Ahmed M. A. Darwish E. S. Ali A. M. (2011). The relationship between motor cortex excitability and severity of Alzheimer’s disease: a transcranial magnetic stimulation study. Neurophysiol. Clin. 41, 107–113. doi: 10.1016/j.neucli.2011.03.002, PMID: 21784322

[ref36] Kobayashi M. Pascual-Leone A. (2003). Transcranical magnetic stimulation in neurology. Lancet 2, 145–156. doi: 10.1016/S1474-4422(03)00321-1 12849236

[ref37] Korzhyk O. Morenko O. Morenko A. Kotsan I. (2019). Gender differences in brain processes during inhibition of manual movements programs. Ann. Neurosci. 26, 4–9. doi: 10.5214/ans.0972.7531.260103, PMID: 31975766 PMC6894627

[ref38] Leisman G. Moustafa A. A. Shafir T. (2016). Thinking, walking, talking: Integratory motor and cognitive brain function. Front. Public Health 4:94. doi: 10.3389/fpubh.2016.00094, PMID: 27252937 PMC4879139

[ref39] Li C. Shan R. Huang C. Constable R. T. Sinha R. (2006). Gender differences in the neural correlates of response inhibition during a stop signal task. Neuroimage 32, 1918–1929. doi: 10.1016/j.neuroimage.2006.05.017, PMID: 16806976

[ref40] Liao D. A. Kronemer S. I. Yau J. M. Desmond J. E. Marvel C. L. (2014). Motor system contributions to verbal and non-verbal working memory. Front. Hum. Neurosci. 8:753. doi: 10.3389/fnhum.2014.00753, PMID: 25309402 PMC4173669

[ref41] Maestú F. de Haan W. Busche M. A. DeFelipe J. (2021). Neuronal excitation/inhibition imbalance: core element of a translational perspective on Alzheimer pathophysiology. Ageing Res. Rev. 69:101372. doi: 10.1016/j.arr.2021.101372, PMID: 34029743

[ref42] Mansouri F. A. Rosa M. G. P. Atapour N. Sigala N. Pardo-Vazquez J. L. Champalimaud F.. (2015). Working memory in the Service of Executive Control Functions SHORT-TERM STORAGE OF INFORMATION REQUIRED TO GUIDE ONGOING OR UPCOMING BEHAVIOR. Front. Syst. Neurosci. 9:166. doi: 10.3389/fnsys.2015.00166, PMID: 26696841 PMC4677100

[ref43] McDonnell M. N. Orekhov Y. Ziemann U. (2006). The role of GABAB receptors in intracortical inhibition in the human motor cortex. Exp. Brain Res. 173, 86–93. doi: 10.1007/s00221-006-0365-2 16489434

[ref44] McGinley M. Hoffman R. L. Russ D. W. Thomas J. S. Clark B. C. (2010). Older adults exhibit more intracortical inhibition and less intracortical facilitation than young adults. Exp. Gerontol. 45, 671–678. doi: 10.1016/j.exger.2010.04.005, PMID: 20417265 PMC2926152

[ref45] Meder A. Liepelt-Scarfone I. Sulzer P. Berg D. Laske C. Preische O.. (2021). Motor cortical excitability and paired-associative stimulation-induced plasticity in amnestic mild cognitive impairment and Alzheimer's disease. Clin. Neurophysiol. 132, 2264–2273. doi: 10.1016/j.clinph.2021.01.011, PMID: 33612394

[ref46] Meeker T. J. Veldhuijzen D. S. Keaser M. L. Gullapalli R. P. Greenspan J. D. (2020). Menstrual cycle variations in gray matter volume, white matter volume and functional connectivity: critical impact on parietal lobe. Front. Neurosci. 14:594588. doi: 10.3389/fnins.2020.594588, PMID: 33414702 PMC7783210

[ref47] Mendoza G. Merchant H. (2014). Motor system evolution and the emergence of high cognitive functions. Prog. Neurobiol. 122, 73–93. doi: 10.1016/J.PNEUROBIO.2014.09.001, PMID: 25224031

[ref48] Mimura Y. Nishida H. Nakajima S. Tsugawa S. Morita S. Yoshida K.. (2021). Neurophysiological biomarkers using transcranial magnetic stimulation in Alzheimer's disease and mild cognitive impairment: a systematic review and meta-analysis. Neurosci. Biobehav. Rev. 121, 47–59. doi: 10.1016/j.neubiorev.2020.12.003, PMID: 33307047

[ref800] Nakamura H. Kitagawa H. Kawaguchi Y. Tsuji H. (1997). Intracortical facilitation and inhibition after transcranial magnetic stimulation in conscious humans. J. Physiol. 498, 817–823. doi: 10.1113/jphysiol.1997.sp021905, PMID: 9051592 PMC1159197

[ref700] Noda Y. Zomorrodi R. Cash R. F. H. Barr M. S. Farzan F. Rajji T. K.. (2017). Characterization of the influence of age on GABAA and glutamatergic mediated functions in the dorsolateral prefrontal cortex using paired-pulse TMS-EEG. Aging (Albany. NY). 9, 556–572. doi: 10.18632/aging.101178, PMID: 28209926 PMC5361681

[ref49] Oldfield R. C. (1971). The assessment and analysis of handedness. Neuropsychologia 9, 97–113. doi: 10.1016/0028-3932(71)90067-4, PMID: 5146491

[ref50] Opie G. M. Ridding M. C. Semmler J. G. (2015). Age-related differences in pre- and post-synaptic motor cortex inhibition are task dependent. Brain Stimul. 8, 926–936. doi: 10.1016/J.BRS.2015.04.001, PMID: 25944419

[ref51] Opie G. M. Rogasch N. C. Goldsworthy M. R. Ridding M. C. Semmler J. G. (2017). Investigating TMS–EEG indices of long-interval Intracortical inhibition at different Interstimulus intervals. Brain Stimul. 10, 65–74. doi: 10.1016/j.brs.2016.08.004, PMID: 27570187

[ref52] Opie G. M. Semmler J. G. (2014). Age-related differences in short- and long-interval intracortical inhibition in a human hand muscle. Brain Stimul. 7, 665–672. doi: 10.1016/j.brs.2014.06.014, PMID: 25088463

[ref53] Opie G. M. Sidhu S. K. Rogasch N. C. Ridding M. C. Semmler J. G. John G. (2018). Cortical inhibition assessed using paired-pulse TMS-EEG is increased in older adults. Brain Stimul. 11, 545–557. doi: 10.1016/j.brs.2017.12.013, PMID: 29317185

[ref54] Páscoa dos Santos F. Verschure P. F. M. J. (2022). Excitatory-inhibitory homeostasis and Diaschisis: tying the local and global scales in the post-stroke cortex. Front. Syst. Neurosci. 15:806544. doi: 10.3389/fnsys.2021.806544, PMID: 35082606 PMC8785563

[ref55] Peña-Casanova J. Casals-Coll M. Quintana M. Sánchez-Benavides G. Rognoni T. Calvo L.. (2012). Estudios normativos españoles en población adulta joven (Proyecto NEURONORMA jóvenes): métodos y características de la muestra. Neurologia 27, 253–260. doi: 10.1016/j.nrl.2011.12.019, PMID: 22397892

[ref56] Pennisi G. Ferri R. Lanza G. Cantone M. Pennisi M. Puglisi V.. (2011). Transcranial magnetic stimulation in Alzheimer’s disease: a neurophysiological marker of cortical hyperexcitability. J. Neural Transm. 118, 587–598. doi: 10.1007/s00702-010-0554-9, PMID: 21207079

[ref850] Peters R. (2006). Ageing and the brain. Postgrad. Med. J. 82, 84–88. doi: 10.1136/pgmj.2005.036665, PMID: 16461469 PMC2596698

[ref57] Redondo-Camós M. Cattaneo G. Perellón-Alfonso R. Alviarez-Schulze V. Morris T. P. Solana-Sanchez J.. (2022). Local prefrontal cortex TMS-induced reactivity is related to working memory and reasoning in middle-aged adults. Front. Psychol. 13:813444. doi: 10.3389/fpsyg.2022.813444, PMID: 35222201 PMC8866698

[ref58] Reitan R. M. Wolfson D. (1985). The Halstead-Reitan neuropsychological test battery: Theory and Interpretattion. Tucson, AZ: Neuropsychology Press.

[ref59] Rezzani R. Franco C. Rodella L. F. (2019). Sex differences of brain and their implications for personalized therapy. Pharmacol. Res. 141, 429–442. doi: 10.1016/j.phrs.2019.01.030, PMID: 30659897

[ref60] Rossi S. Antal A. Bestmann S. Bikson M. Brewer C. Brockmöller J.. (2021). Safety and recommendations for TMS use in healthy subjects and patient populations, with updates on training, ethical and regulatory issues: expert guidelines. Clin. Neurophysiol. 132, 269–306. doi: 10.1016/j.clinph.2020.10.003, PMID: 33243615 PMC9094636

[ref61] Rossini P. M. Burke D. Chen R. Cohen L. G. Daskalakis Z. Di Iorio R.. (2015). Non-invasive electrical and magnetic stimulation of the brain, spinal cord, roots and peripheral nerves: basic principles and procedures for routine clinical and research application: an updated report from an I.F.C.N. Committee. Clin. Neurophysiol. 126, 1071–1107. doi: 10.1016/j.clinph.2015.02.001, PMID: 25797650 PMC6350257

[ref62] Rubia K. Lim L. Ecker C. Halari R. Giampietro V. Simmons A.. (2013). Effects of age and gender on neural networks of motor response inhibition: from adolescence to mid-adulthood. Neuroimage 83, 690–703. doi: 10.1016/j.neuroimage.2013.06.078, PMID: 23845427

[ref900] Salat D. H. Kaye J. A. Janowsky J. S. (2001). Selective preservation and degeneration within the prefrontal cortex in aging and Alzheimer disease. Arch. Neurol. 58, 1403–1408. doi: 10.1001/archneur.58.9.1403, PMID: 11559311

[ref63] Sanger T. D. Garg R. R. Chen R. (2001). Interactions between two different inhibitory systems in the human motor cortex. J. Physiol. 530, 307–317. doi: 10.1111/J.1469-7793.2001.0307L.X, PMID: 11208978 PMC2278414

[ref64] Schicktanz N. Schwegler K. Fastenrath M. Spalek K. Milnik A. Papassotiropoulos A.. (2014). Motor threshold predicts working memory performance in healthy humans. Ann. Clin. Transl. Neurol. 1, 69–73. doi: 10.1002/acn3.22, PMID: 25356384 PMC4207507

[ref65] Schmidt M. (1996). Rey auditory and verbal learning test: A handbook. Los Ángeles: Western Psychological Services.

[ref66] Schmidt-Wilcke T. Fuchs E. Funke K. Vlachos A. Müller-Dahlhaus F. Puts N. A. J.. (2018). GABA—from inhibition to cognition: emerging concepts. Neuroscientist 24, 501–515. doi: 10.1177/1073858417734530, PMID: 29283020

[ref67] Shibuya K. Park S. B. Geevasinga N. Huynh W. Simon N. G. Menon P.. (2016). Threshold tracking transcranial magnetic stimulation: effects of age and gender on motor cortical function. Clin. Neurophysiol. 127, 2355–2361. doi: 10.1016/j.clinph.2016.03.009, PMID: 27178853

[ref68] Sohal V. S. Rubenstein J. L. R. (2019). Excitation-inhibition balance as a framework for investigating mechanisms in neuropsychiatric disorders. Mol. Psychiatry 24, 1248–1257. doi: 10.1038/s41380-019-0426-0, PMID: 31089192 PMC6742424

[ref69] Sukenik N. Vinogradov O. Weinreb E. Segal M. Levina A. Moses E. (2021). Neuronal circuits overcome imbalance in excitation and inhibition by adjusting connection numbers. Proc. Natl. Acad. Sci. U. S. A. 118:e2018459118. doi: 10.1073/pnas.2018459118, PMID: 33723048 PMC8000583

[ref70] Takahashi S. Ukai S. Kose A. Hashimoto T. Iwatani J. Okumura M.. (2013). Reduction of cortical GABAergic inhibition correlates with working memory impairment in recent onset schizophrenia. Schizophr. Res. 146, 238–243. doi: 10.1016/j.schres.2013.02.033, PMID: 23523695

[ref71] Terunuma M. Revilla-Sanchez R. Quadros I. M. Deng Q. Deeb T. Z. Lumb M.. (2014). Postsynaptic GABAB receptor activity regulates excitatory neuronal architecture and spatial memory. J. Neurosci. 34, 804–816. doi: 10.1523/JNEUROSCI.3320-13.2013, PMID: 24431439 PMC3891960

[ref72] Travica N. Ried K. Hudson I. Sali A. Scholey A. Pipingas A. (2020). Gender differences in plasma vitamin C concentrations and cognitive function: a pilot cross-sectional study in healthy adults. Curr. Dev. Nutr. 4:nzaa038. doi: 10.1093/CDN/NZAA038, PMID: 32337476 PMC7170048

[ref73] Valls-Solé J. Pascual-Leone A. Wassermann E. M. Hallett M. (1992). Human motor evoked responses to paired transcranial magnetic stimuli. Electroencephalogr. Clin. Neurophysiol. Evoked Potentials 85, 355–364. doi: 10.1016/0168-5597(92)90048-G 1282453

[ref74] Versace V. Sebastianelli L. Ferrazzoli D. Romanello R. Ortelli P. Saltuari L.. (2021). Intracortical GABAergic dysfunction in patients with fatigue and dysexecutive syndrome after COVID-19. Clin. Neurophysiol. 132, 1138–1143. doi: 10.1016/j.clinph.2021.03.001, PMID: 33774378 PMC7954785

[ref75] Vlachou S. (2022). GABAB receptors and cognitive processing in health and disease. Curr. Top Behav. Neurosci. 52, 291–329. doi: 10.1007/7854_2021_231, PMID: 34382179

[ref76] Wechsler D. (2012). “WAIS-IV. Escala de inteligencia de Wechsler para adultos-IV” in Manual De Aplicación Y Corrección (Madrid: NCS Pearson, Inc.)

[ref77] Wiley J. Jarosz A. F. (2012). How working memory capacity affects problem solving. Psychol. Learn. Motiv. Adv. Res. Theory 56, 185–227. doi: 10.1016/B978-0-12-394393-4.00006-6

[ref78] Zadey S. Buss S. S. McDonald K. Press D. Z. Pascual-Leone A. Fried P. J. (2021). Higher motor cortical excitability linked to greater cognitive dysfunction in Alzheimer's disease: results from two independent cohorts. Neurobiol. Aging 108, 24–33. doi: 10.1016/j.neurobiolaging.2021.06.007, PMID: 34479168 PMC8616846

